# Effect of peer videorecording feedback CPR training on students’ practical CPR skills: a randomized controlled manikin study

**DOI:** 10.1186/s12909-022-03563-9

**Published:** 2022-06-22

**Authors:** Lian Lin, Shaozhou Ni, Yaqi Liu, Jingyi Xue, Binyu Ma, Dan Xiong, Yan Zhao, Xiaoqing Jin

**Affiliations:** 1 Emergency Center, Zhongnan Hospital of Wuhan University, 169 Donghu Road, Hubei 430071 Wuhan, China; 2 Hubei Clinical Research Center for Emergency and Resuscitation, Zhongnan Hospital of Wuhan University, 169 Donghu Road, Wuhan, 430071 Hubei China; 3 The Second Clinical College of Wuhan University, Wuhan, 430071 Hubei China

**Keywords:** Cardiopulmonary resuscitation, Videorecording, Feedback, Manikin

## Abstract

**Background:**

The aim of this study was to compare one-month acquisition and half-a-year quality retention of cardiopulmonary resuscitation (CPR) skills after initial training of medical students between peer videorecording feedback training (PVF) and traditional peer verbal feedback training (TVF).

**Methods:**

Participants were randomly assigned to the PVF group (*n* = 62) and the TVF group (*n* = 65). All participants received a 45-min CPR training program performed by an American Heart Association basic life support-certified instructor, and then they began two hours of practice in groups. During interactive peer learning, students cooperated in couples of a doer and a helper to realize maximization of each other’s learning. In the PVF group, training performance feedback came from peers based on practice videorecording. In the TVF group, feedback came from peers verbally without videorecording. CPR quality was tested at 1 and 6 months after training.

**Results:**

After 1 month of initial training, the PVF group had a better presentation of CPR skills acquisition than the TVF group. Compared to the TVF group, the PVF group had significantly higher total scores, compression depth, appropriate compression depth, and complete chest recoil (*p* < 0.05). Moreover, compression interruption was a significantly positive change in the PVF group compared to the TVF group (*p* < 0.05). However, after 6 months, proportions of appropriate compression depth in the PVF group were better than those in the TVF group (*p* < 0.05). The differences in total scores, compression depth, appropriate compression depth, complete chest recoil and compression interruption were non-significant (all *p* > 0.05).

**Conclusions:**

Compared to TVF, PVF is more effective in enhancing CPR skill acquisition at 1 month. After half a year, CPR skill quality was obviously reduced in both groups, and no difference in CPR quality was found between the two groups.

## Introduction

Out-of-hospital cardiac arrest (OHCA) remains a leading cause of death. There are approximately 55 OHCA cases per 100,000 people worldwide, with a high incidence but a low survival rate; approximately 7 percent of patients survive [1]. High-quality cardiopulmonary resuscitation (CPR) is connected with significantly enhanced survival rates for OHCA in human and animal studies [2, 3]. According to the international consensus, a bystander who has received CPR training is the most significant element to rescue a patient with sudden cardiac arrest in an out-of-hospital setting [4].

An essential component of students’ CPR training is feedback: students need to be able to distinguish what they did well or badly, what else they have to do, and so on. A systematic review identified that feedback is an important feature of simulation-based medical education [5]. Feedback urges rethinking and encourages students to enhance their skills. During traditional CPR training, feedback is always transmitted verbally [6]. However, there is a threat that when students focus on other subjects of the course, verbal feedback may be forgotten, because in most situations they are unlikely to have a chance to use their CPR skills soon after training. In recent years, videorecording has been used in CPR training as a technique for assessing CPR quality [7]. Videorecording is a helpful instrument for offering feedback, as students can observe their own performance.

Several studies have proved that peer-led (students teaching their student peers) CPR training is an advantageous way to teach high-quality CPR [8, 9]. Peer teaching can promote effective training by building smaller reciprocal groups with encouragement and enthusiasm among peers [10]. At the same time, peer feedback provides students with opportunities to learn from each other, which can stimulate students’ interest in learning and improve the quality and effectiveness of training.

It would be beneficial to integrate videorecording and reciprocal peer learning feedback to optimize qualitative assessment of CPR performance. The purpose of this study was to compare the 1-month effect of training quality and 6 months retention ability of CPR skills between peer videorecording feedback training (PVF) and the traditional peer verbal feedback (TVF) method on manikins to develop a better way to acquire high-quality CPR skill.

## Methods

### Participants and setting

The ethics committee of Zhongnan Hospital of Wuhan University (Wuhan, Hubei, China) approved this study, and all participants provided written consent. All procedures were performed in accordance with relevant guidelines. The study was conducted between March 16, 2021 and September 10, 2021 at the Second Clinical School, Wuhan University (Wuhan, Hubei, China). Fourth-year medical students were enrolled in this study. Background information of participants including age, gender, weight, height, and body mass index (BMI) was recorded. All participants previously had no CPR training or experience. Participants with physical illness that might affect normal capacity for action were excluded, such as vertigo, pneumonia and fracture.

### Study design

#### Training program

A 45-min CPR training program was performed by American Heart Association (AHA) basic life support-certified instructors, which was in accordance with the 2020 AHA CPR guidelines. A 45-min CPR training program included the following: (1) Check responsiveness, yell for help, activate the emergency response system, make an emergency call for help, and send for an AED; (2) Check breathing and pulse for at least 5 s and no more than 10 s; and (3) CPR instruction and practice.The training program included showing CPR rudimentary knowledge and skills with a PowerPoint exhibition (Microsoft Corporation, Redmond, WA, USA), playing video of single-rescuer CPR skills, and showing fundamental operations on manikins. All the instructors were informed about the grouping assignment, and they did not join in the grouping assignment.

#### Group and CPR practice

**Fig. 1:**
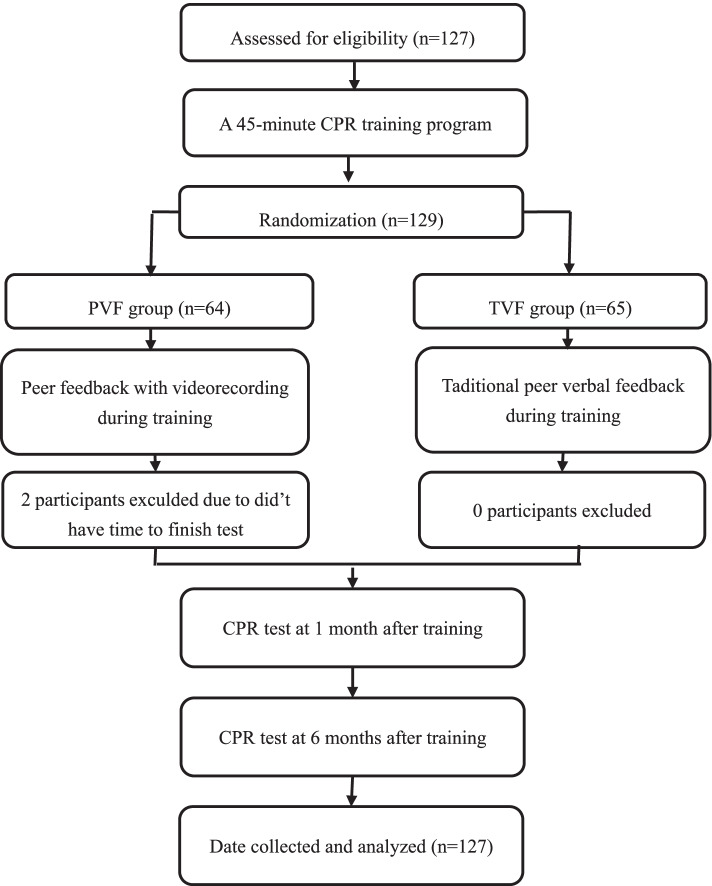
Participant flow chart. CPR: cardiopulmonary resuscitation, PVF: peer videorecording feedback, TVF: traditional peer verbal feedback

Students were randomly assigned to the PVF group (*n* = 64) or the TVF group (*n* = 65) using a card in a sealed envelope. If “P” was on the card in the envelope, the participants were assigned to the PVF group, while if “T” was on the card in the envelope, the participants were assigned to the TVF group. However, 2 participants in PVF group exculded due to their not having had time to complete the test. Students were allocated into pairs randomly. During interactive peer learning, students cooperated in couples of a doer and a helper. While a helper was directing, observing, and giving performance-related feedback based on the doer behavior during training, the doer was performing CPR. In the PVF group, one student was performing; meanwhile, the peer was shooting a video with a smartphone, and then they switched roles. Then, they watched their practice on their own phone and the peer also provide feedback based on the video. However, in the TVF group, training feedback information was transmitted to the peer only verbally. The two groups were distributed into different operation rooms, and practiced on adult Laerdal Resusci Anne QCPR torso manikins (Laerdal China Ltd., Hangzhou, China). All participants took CPR tests after 1 and 6 months of initial training. The tests were provided with a simulated scene of onlooking an adult collapse outside the hospital. The tests included five cycles of single-rescuer CPR in an imitated situation using QCPR manikins; each cycle of chest compressions: ventilation was 30:2. Study protocol was summarized in Fig. 1.

### Sample size calculation

According to our preliminary trial, we estimated that PVF would result in 70% in correct compression depth proportion at 1 month after training. We calculated that it would require at least 64 participants in each group to detect the important difference of 20% in the correct compression depth proportion between PVF group (70%) and the TVF group (90%) with a power of 0.8, and a type I error of 0.05. Finally, 129 participants were included in the present study.

### Outcome measures

The Laerdal SimPad PLUS was linked to the QCPR manikin, and it mechanically recorded the following variables: total scores, chest compression (CC) rate, CC depth, the percentage of appropriate CC rate (100–120/min), the percentage of appropriate depth (50–60 mm), proportion of complete chest recoil (%), proportion of correct hand location position, mean percentage of ventilations with adequate volume, and CC interruption time. Participants’ CPR performance total scores (range: 0–100) were generated from the SimPad system report. CPR skill was tested at 1 and 6 months after initial training. Appropriate CC depth, rate, correct hand location position, and complete chest recoil were confirmed according to the 2015 American Heart Association (AHA) guidelines.

### Statistical analysis

Data were analyzed using commercial software (SPSS version 19, IBM Corporation, New York, USA). Data were expressed as the means ± SD, percentages, or numbers. Comparisons across groups were conducted using the chi-square test for categorical variables. Non-parametric continuous variables were analyzed by the Mann–Whitney U test, and parametric continuous variables were analyzed by Student’s *t* test. Assessment of the distributions of the variables was conducted by Levene’s test for homogeneity of variances. *P* < 0.05 was considered significant.

## Results

### Characteristics of participants

The participant flow chart is shown in Fig. 1. Among 127 participants, 65 were randomly assigned to the TVF group, and 62 were randomly assigned to the PVF group. No students were excluded before randomization because of health problems. The demographic characteristics of students in the two groups are summed up in Table 1; the percentage of females was 58% in the TVF group and 53% in the PVF group (*p* = 0.55). In addition, there were no statistical differences in height, weight, or BMI between groups (*p* > 0.05).

**Table 1:** Characteristics of participants

	TVF (*n* = 65)	PVF (n = 62)	*p*
Gender			
Male	27(42%)	29(47%)	0.556
Female	38(58%)	33(53%)	
Height (cm)	166.7 ± 8.2	166.0 ± 7.7	0.719
Weight (kg)	60 ± 11	59 ± 10	0.446
BMI	21.5 ± 3.0	21.0 ± 2.4	0.446

Data are expressed as number (percentage) or mean ± SD

*PVF* peer videorecording feedback, *TVF* traditional peer verbal feedback, *BMI* body mass index

### CPR skill acquisition after 1 month

The CPR performance after 1 month was retested, as shown in Table 2. Compared to the TVF group, the PVF group had a significant improvement in total scores (TVF vs PVF: 69.6 ± 21.4 vs 81.2 ± 16.0, *p* = 0.001). In the PVF group, there was an obviously better performance in compression depth (53.8 ± 5.9 vs 56.2 ± 4.0, *p* = 0.028) and appropriate compression depth (79.7 ± 30.0 vs 90.6 ± 16.1, *p* = 0.013), and proportions of complete chest recoil (84.6 ± 22.1 vs 93.0 ± 12.7, *p* = 0.027). Moreover, compression interruption (12.7 ± 3.2 vs 11.6 ± 2.3, *p* = 0.027) showed a positive change in the PVF group compared to TVF group. However, CPR quality outcome measures, such as compression rate, appropriate compression rate, correct hand position, and percentage of ventilations with adequate volume, were not statistically significant between the two groups.

**Table 2:** CPR quality retention after 1 month

	TVF (*n* = 65)	PVF (*n* = 62)	*p*
Total scores (%)	69.6 ± 21.4	81.2 ± 16.0	0.001
Compression depth (mm)	53.8 ± 5.9	56.2 ± 4.0	0.028
Appropriate compression depth (%)	79.7 ± 30.0	90.6 ± 16.1	0.013
Compression rate (cpm)	110.5 ± 10.2	110.3 ± 7.8	0.910
Appropriate compression rate (%)	64.5 ± 32.0	73.8 ± 26.6	0.139
Correct hands position (%)	96.4 ± 12.4	95.8 ± 17.0	0.076
Complete chest recoil (%)	84.6 ± 22.1	93.0 ± 12.7	0.027
Compressions interruption (s)	12.7 ± 3.2	11.6 ± 2.3	0.027
Percentage of ventilations with adequate volume (%)	36.7 ± 37.6	40.9 ± 39.6	0.634

Appropriate chest compression rate, depth, correct hand position, and complete chest recoil were defined according to the 2015 American Heart Association (AHA) guideline

*PVF* peer videorecording feedback, *TVF* traditional peer verbal feedback

### CPR quality retention after half a year

After half a year, 65 students in the TVF group and 62 students in the PVF group were assessed, as summarized in Table 3. The quality of CC was significantly decreased after half a year as compared with 1-month performance in both the TVF and PVF groups. As showed in Table 3, the proportions of appropriate compression depth (TVF vs PVF, 60.9 ± 39.4 vs 70.1 ± 37.1, *p* = 0.036) in the PVF group were better compared with those in the TVF group. However, no significant differences were observed in the other outcome measures. In summary, all these results suggested that CPR skill quality retention was obviously decayed in both groups, and there were little statistically significant differences between the two groups after half a year.

**Table 3:** CPR quality retention after 6 months

	TVF (*n* = 65)	PVF (*n* = 62)	*p*
Total scores (%)	55.1 ± 23.2	55.3 ± 25.4	0.990
Compression depth (mm)	52.4 ± 7.0	49.8 ± 8.2	0.065
Appropriate compression depth (%)	60.9 ± 39.4	70.1 ± 37.1	0.036
Compression rate (cpm)	106.3 ± 12.3	103.1 ± 10.3	0.166
Appropriate compression rate (%)	41.9 ± 35.2	49.2 ± 35.6	0.311
Correct hands position (%)	89.2 ± 27.0	87.0 ± 28.7	0.908
Complete chest recoil (%)	88.2 ± 23.2	83.9 ± 25.6	0.247
Compressions interruption (s)	16.8 ± 6.9	17.3 ± 7.5	0.791
Percentage of ventilations with adequate volume (%)	52.4 ± 34.7	59.3 ± 37.7	0.272

Appropriate chest compression rate, depth, correct hand position, and complete chest recoil were defined according to the 2015 American Heart Association (AHA) guideline

*PVF* peer video feedback, *TVF* traditional peer verbal feedback

## Discussion

In this prospective observational study, we investigated the training effects of PVF training on CPR quality compared with TVF training. We found that the PVF group had a better presentation of CPR skill acquisition than the TVF group, and there was little distinction in half-a-year quality retention. These findings showed that there was an obvious advantage of peer feedback combined with videorecording, which was a good method of CPR training, when compared to the TVF method after training 1 month later.

Our study derived resemblance conclusions with the research by Spence, A.D., et al. [11]; they found that the video feedback group had a significantly greater increase in the total score compared to the traditional verbal feedback group after training 1 month later. However, in the study by Spence, A.D., et al., decay of CPR skills and knowledge was not researched further to confirm its long-term consequence. In the present study, we found there was little distinction in half-a-year CPR skill quality retention between the PVF group and TVF group. Anantasit, N., et al. also derived resemblance conclusions with our study; they found that videorecording feedback during individual CPR skills can increase six-week CPR skill acquisition, resulting in higher-quality CPR performance [12]. The CC quality of the two groups in our study was significantly diminished after half-a-year compared to 1 month, and for this there maybe multiple reasons. Above all, these results may be due to simple declinein CPR technique and knowledge; several studies [13–15] have found that CPR skills decreased along with time without recurring practice. To reduce the decline in CPR skills, training every 1 month might alleviate skill decay, but it is uncertain whether this will lead to better outcomes, if training is refreshed 2, 3, 4, or 5 months. Further data are required to evaluate decline across these time periods. In addition, the decay of CPR skills may be due to the training method, and it’s possible that videorecording feedback training might merely improve the short-term ability, while not really contributing contribute to long-term retention. The decay time of CPR skills after peer videorecording feedback training needs to be investigated further.

Peer feedback provides students with opportunities to learn from each other, and there may even be competition among students, which might promote students’ interest in learning and improving the quality of training [16]. However, we can only focus on limited information for this one study; compared to PVF, TVF may leave out some vital information, and comprehensive feedback cannot be delivered to another peer [17]. The lack of evaluation criteria of verbal feedback provided by peers are limitations that may have impacted CPR skill acquisition [18].

Videorecording is a helpful instrument for offering feedback, as students can observe their own performance. Their memories of simulation training and verbal feedback received promptly afterward were inadequate, and repeated videorecording revision made them efficient at distinguishing better and worse practice with more proficiency and allowed them to provide more comprehensive feedback on their own and peers’ practice [19]. Students found that it was easier to provide more specific feedback when given the opportunity to watch the videorecording immediately after practice [11].

PVF is a better method of motivating reflection: students have an opportunity to consider their own performance and distinguish good and bad practice. An advantage of PVF is that the student can revise it more than once, thereby reinforcing knowledge and skill [11]. Students in PVF can review their own and peer training videos and discuss their performance, which may provide more comprehensive feedback than that given in the TVF group [20]. Overall, PVF is an effective way to identify the student’s basic skill level which is a quite useful tool as training feedback for the students.

## Limitations

There are some limitations to this study. First, although we used manikin simulation training, we did not examine the CPR skill under real clinical resuscitations. The experience of compressions, and the controls and power diverge to a certain degree between manikins and humans, and this transformation may have affected the students’ CPR skill performances. Second, the objects of the study were medical students, and we believe that CPR performances among different populations, such as the public, need also to be assessed.

## Conclusion

This observational study suggested that the PVF group CPR performance was obviously better than that with TVF after training for 1 month, with a significant improvement in total scores, compression depth, appropriate compression depth, proportions of complete chest recoil, and compression interruption. Compared to TVF, PVF was more effective in enhancing CPR skill acquisition. After six months, CPR skill quality retention was obviously reduced in both groups, and there was little significant difference between the two groups.

## Data Availability

The datasets used and/or analyzed during the current study are available from the corresponding author on reasonable request.

## Abbreviations

CPR: Cardiopulmonary Resuscitation
PVF: Peer Videorecording Feedback training
TVF: Traditional peer Verbal Feedback training
OHCA: Out-of-Hospital Cardiac Arrest
BMI: Body Mass Index
AHA: American Heart Association
CC: Chest Compression
